# Inquiring about Loss Aversion of Achievement Value

**DOI:** 10.3390/bs13050400

**Published:** 2023-05-10

**Authors:** Chung-Chin Wu

**Affiliations:** Department of Early Childhood Education, National Pingtung University, Pingtung 900391, Taiwan; minin72704@mail.nptu.edu.tw

**Keywords:** achievement motivation, intrapersonal comparison, interpersonal comparison, loss aversion, prospect theory, self-discrepancy theory

## Abstract

According to the achievement motivation theory, in achievement context, students may have to not only approach success/gain (e.g., strive to get a better grade) but also avoid failure/loss (e.g., avoid performing worse). However, these two types of achievement motivation have often been investigated separately. In contrast, loss aversion, a central argument in prospect theory, posits that avoiding losses has a greater impact on preferences than does approaching gains; suggesting that gain approach and loss avoidance should be treated as asymmetric forces that can be analyzed simultaneously to study approach to gain and avoidance to loss among students in terms of grades. The main purposes of this study were to propose an alternative measure to frame the dynamic evaluation process in the context of achievement that considers students’ sensitivity to performance change, and to further investigate students’ loss aversion in relation to grades through intrapersonal and interpersonal comparisons. A total of 41 and 72 college students participated in study 1 and study 2, respectively. One-way repeated measure ANOVA was conducted for the former sample while the single sample *t*-tests and independent sample ANOVA were used for the latter. Through the implementation of this alternative measure, the results revealed that (1) college students were more sensitive to performance changes than to their current or final performance, and (2) loss aversion was dependent on the referents. Students were averse to interpersonal loss, but not to intrapersonal loss. These findings indicate the usefulness of the proposed measure for investigating the asymmetric responses between two types of achievement motivation, and the proposed measure can be used to extend and revise the explanatory boundaries of prospect theory and self-discrepancy theory.

## 1. Introduction

Loss aversion, which is a core proposition in prospect theory, reflects an asymmetry of value. It refers to change that makes things worse (loss) or leads to disadvantages; such circumstances have a greater impact on preferences than do gains or improvements [1,2]. It suggests that people may not feel the same towards a certain final state that involved different changes in state. For example, say there are two people who currently have $100, but yesterday, one owned $1000 while the other had $10. The former may feel bad about the relative loss, but the latter may feel happy about the relative gain. It suggests that students may not feel the same about a certain final grade that involves different changes in grade. Moreover, it is not clear whether students are more sensitive to changes in grade than their final grade. By identifying this effect, it may be helpful for teachers to arrange an activity (e.g., ask students to evaluate their relative performances) to evoke their self-regulation behaviors and expectations to pursue better academic performance.

Loss aversion is calculated by dividing the amount of gain that someone needs to compensate for the negative feelings associated with a certain amount of loss by the loss. According to loss aversion, people do not require the same amount of gain for their corresponding loss. For example, sellers may expect to receive $100 gain from a buyer for selling (loss) a mug that only costs $50. It has been extensively demonstrated that negative feelings toward loss are greater than positive feelings toward the corresponding gain. The resulting loss aversion ratio is approximately 2:1 [3,4]. However, it has been demonstrated that this ratio decreases or reverses for small loss [5,6], and decreases with education and individualism [6,7]. Researchers have reviewed empirical evidence and concluded that evidence from real-world phenomena provides little support for loss aversion [8]. This negative evidence has primarily been observed in the context of decision-making behaviors connected with money or the trade of goods. It remains unclear whether loss aversion could help explain the asymmetric predisposition of achievement motivation in the achievement context. In order to fill this research gap, this study investigated how students respond to losses and gains in grades or points, which do not involve a physical trade-off (money or goods). The following hypotheses were proposed:

**H1:**  *Students are more sensitive to changes than to a certain final state*.

In the achievement context, gains and losses in grades or points are important incentives that drive two types of achievement motivation: approach and avoidance motivations. The former and the latter orients individuals to approach positive and to avoid negative possibilities/events, respectively. As researchers note, approach and avoidance motivation operate simultaneously, and students struggle to avoid failure rather than work toward success in the achievement context [9]. This suggests that the psychological forces linked to approach motivation (driven by a gain in points) and avoidance motivation (oriented by loss in points) may be asymmetric. It was hypothesized that the resulting loss aversion ratio would be approximately 2:1. For example, students may expect to earn 10 points more than what they expected on an upcoming exam to compensate for the negative feelings that come with earning 5 points less than their normal score on a current test.

In achievement motivation, approach motivation may be elicited by a gain in points when a student’s present or expected performance is better than it was in the past or they outperform their peers. On the other hand, avoidance motivation may be triggered by loss in points when a student’s present or expected performance is worse than it was in the past or inferior to other students. As a result, there are two types of loss (failure) that result from intrapersonal and interpersonal comparisons. Students may encounter two conditions: intrapersonal loss and interpersonal loss. For example, in the first condition, students compare the 80 points that they received on a midterm (i.e., current performance) to the 85 points that they usually average on class quizzes (i.e., what they have done in the past). In the second condition, students compare the 85 points that they receive on a midterm (i.e., current performance) to the 90 point class average on the midterm (i.e., the normative performance of others). However, with respect to loss aversion, it is currently unclear whether students are equally averse toward these two types of losses.

According to the self-discrepancy theory, there are three types of selves: actual self, ought self, and ideal self. In the current study, real performance reflects actual self, and ought self is reflected in the average performance that students demonstrated in the past (i.e., their normal level of performance). Expected performance in an upcoming event (i.e., an exam) represents the ideal self. Previous research has found that discrepancies between the actual self and the ought self may cause negative outcomes, such as social avoidance and fear of negative evaluation [10]. Others have found that discrepancy between actual self and ought self negatively predicts attentiveness, but does not predict or correlate with other negative effects, such as fear and sadness [11], which contradicts the predictions offered by the self-discrepancy theory. This seems to suggest that students may not feel dissatisfied when their present performance is lower than their past achievement. It is more reasonable to predict that students may feel a bit dissatisfied with intrapersonal loss if a similar measurement is used. Consequently, the following hypothesis was proposed:

**H2:**  *Students feel a bit dissatisfied when their current performance is inferior to their normal level of achievement*.

This study also investigated whether possible dissatisfaction linked to intrapersonal loss could be compensated by gain attained from interpersonal comparison. For example, if a student experiences negative emotions as a result of receiving 5 points less on a test than they usually do, this could be compensated if that latest score is 5 points higher than the class average. According to the achievement goal theory and its empirical findings, students may feel satisfied or proud when their current performance is better than the performance of others [12]. It was expected that the negative feelings linked to intrapersonal loss would be compensated by interpersonal gains. Consequently, the following hypothesis was proposed:

**H3:**  *Students’ dissatisfaction with intrapersonal loss is compensated and even overturned by an increasing amount of interpersonal gains*.

In addition, loss aversion in the prospect theory does not predict whether students are equally averse to intrapersonal and interpersonal losses. According to empirical testing of the self-discrepancy theory, a negative affect (i.e., disappointment, dissatisfaction, and sadness) only occurs when there is a discrepancy between an individual’s actual self and ideal self [10,11]. It has been argued that students are more likely to fear being judged as incompetent if they do worse than others (e.g., 5 points lower than others or a normal standard) [9,13,14]. This suggests that students may be averse to intrapersonal loss when their current performance is lower than their expected or ideal performance, and they may hope to receive a grade that is higher than their peers in order to compensate for this kind of intrapersonal loss. This results in an asymmetric intrapersonal loss aversion ratio. Moreover, compared with intrapersonal loss, students are more averse to interpersonal losses when their current performance is lower than the class average. It may be that the resulting interpersonal loss aversion ratio corresponds to typical findings in the Economics field. The following hypotheses were thus proposed:

**H4:**  *The intrapersonal loss aversion ratio is significantly higher than l but significantly lower than 2*.

**H5:**  *The interpersonal loss aversion ratio is significantly higher than l and not significantly different from 2*.

**H6:**  *Students are more averse to interpersonal loss than intrapersonal loss*.

### Purposes

In the achievement context, it remains unclear whether students are more sensitive to changes in grades than to their final grade, as is proposed in the prospect theory in the Economics field. The methods used in traditional studies are unable to be used to investigate this dynamic evaluation process among students and the asymmetry between approach gain and avoid losses in relation to grades. In addition, the loss aversion proposed in the prospect theory does not predict if individuals may be averse to losses involving different referents (i.e., gain and loss resulting from intrapersonal or interpersonal comparisons). Meanwhile, the achievement goal agrees with the simultaneous operation of approach gains and avoid losses with respect to different referents, but does not assume and clarify the asymmetry between these two forces. However, the achievement goal suggests how individuals may feel about losses, but does not clarify how individuals may feel about intrapersonal gains and losses. The self-discrepancy theory, however, does. Consequently, the alternative measure proposed in this study is based on the integration of these three theories to examine loss aversion in relation to grade invoving intrapersonal and interpersonal comparisons to assess the transferability of loss aversion from the Economics field to the achievement context, and to clarify whether students were equally averse to intrapersonal and interpersonal losses. The explanatory boundaries of these theories can be confirmed and may be extended through clarifying the issues above. The purposes of this study were two-fold:

(1) to clarify if students are more sensitive to changes than to a certain final state (H1, H2, and H3 are related to this purpose);
(2) to examine how averse students are to intrapersonal and interpersonal losses (H4, H5, and H6 are related to this purpose).

## 2. Methodology

### 2.1. Participants

In total, 41 and 72 college students consented to participate in study 1 and study 2, respectively. Forty-one college students (22 males and 19 females, mean age = 18.71, *SD* = 0.31) answered the same four questions delineated four conditions in study 1, while 72 students randomly divided into two groups (36 in group one and 36 in group two) answered one of two questions, each of which described two conditions in study 2 (36 male and 36 female, mean age = 19.56, *SD* = 0.26).

### 2.2. Measures and Procedure

Four questions were asked in study 1, which were mainly used to test H1, H2, and H3, and served as a basis for testing differential loss aversion in study 2. 

In study 1, the first two questions delineated intrapersonal comparison, while the remaining two involved both intra- and inter-personal comparison (mixed comparison). They were used to assess if students were more sensitive to changes in points than to a certain final state, and students’ answers to these questions signaled whether to probe further into differential loss aversion. After answering the four questions in study 1, students were told that the mean of the average points that they received on class quizzes and the midterm constituted their final grade, and they were asked “How satisfied were you with your performance?” at the end of each question (participants had five options to choose from: extremely unsatisfied, a little unsatisfied, acceptable, a little satisfied, and extremely satisfied).

In study 1, the first question was designed to set a baseline attitude for success (intrapersonal 5-point gain). It was described in the following way:

Average points on class quizzes: 80 points on subject A

Midterm points: 85 points on subject A

The second question described an identical gain (5-point gain from 80 to 85 points) in subject A that was accompanied by a 5-point loss (from 85 points on average for class quizzes to 80 points on the midterm) in subject B. It was described in the following way:

Average points on class quizzes: 80 points on subject A, 85 points on subject B

Midterm points: 85 points on subject A, 80 points on subject B

The same average points received on class quizzes and the midterm in subject A in the first question were used in the second question. In both subjects, the expected average points on class quizzes and the midterm was 82.5 in the second question and identical to the final midterm average score in question 1. If intrapersonal loss did not exert an effect on psychological feeling, it was assumed that participants showed no differences in satisfaction level between the two questions. In contrast, according to loss aversion, participants were hypothesized to show lower levels of satisfaction over their final midterm performance if they viewed it as an intrapersonal loss. 

The third and fourth questions were mixed comparison conditions in which the intrapersonal loss frame was accompanied by 5- and 10-point gain frames, respectively. These questions were designed to clarify whether the feeling of identical intrapersonal loss (5 points) that was reflected in response to the second question could be affected by interpersonal gains. The same average point score and midterm point score used in the second question appeared in the third and the fourth questions, and the final average score in these two questions was also identical to the one used in the first and second questions. The third and the fourth questions were described as follows:

Average points on class quizzes: 85 points

Midterm points: 80 points

Class average points on midterm: 75 points (for the third)/70 points (for the fourth)

Theoretically, students should show no differences in their levels of satisfaction when responding to the four questions if they only focus on the average final grade rather than relative gains and losses, because the four questions have the same average final grade (82.5). The following conditions were identified as supporting evidence for H1, H2, and H3:

(1) H1 could be supported if there were differences in levels of satisfaction among the four questions, which would demonstrate that students were more sensitive to changes than a certain final state.
(2) The mean for question 2 was significantly higher than it was for question 1, which could offer supporting evidence for H2.
(3) The mean for question 3 and 4 was significantly higher than it was for questions 2 and 3, which could offer supporting evidence for H3.

In study 2, two questions were designed to examine whether students were averse in different ways to intrapersonal and interpersonal losses. The first and the second questions were used to test H4 and H5, respectively, and the results derived from these two questions were together to test H6. The first question was designed to examine how averse students were to intrapersonal loss and was used to test H4:

Midterm expected points: 92 points in subject A, 82 in subject B

Midterm actual points: 87 points in subject A, ____ n subject B

In the first question, intrapersonal loss in subject A was presented before the points in subject B. Students were told that they could expect to receive 92 points in subject A, but that they actually only earned 87 points in that subject. They were also told that the midterm exam of subject B was going to be held tomorrow and that they could expect to receive 82 points on that particular test. They were then asked to write down the minimum number of points that they could receive in subject B to compensate for their losses in subject A. 

The second question was designed to examine how averse students were to interpersonal loss and was used to test H5:

Midterm expected points: 82 points in subject A, 82 in subject B

Midterm actual points: 87 points in subject A (class average was 92 points) ____ in subject B

In the second question, interpersonal gain accompanied by interpersonal loss in subject A was presented ahead of the points in subject B. Students were told that they could expect to earn 82 points in subject A but ultimately received 87 points (interpersonal gain) and that the class average was 92 points (interpersonal loss). They were also told that the midterm exam of subject B would be held tomorrow and that they could expect to earn 82 points on that test. Students were then asked to write down the minimum number of points that they would need to get in subject B to compensate for a 5 points loss in subject A.

Loss aversion ratios for each individual who respectively answered these two questions were identified by dividing the relative gain in points in subject B to the loss amount in subject A. 

### 2.3. Data Analysis

One-way repeated measure ANOVA was conducted in study 1 to examine how sensitive students were to changes. Single sample *t*-tests and independent sample ANOVA were applied in study 2. In study 1, the significant main effect served as supporting evidence for H1. Significant results from post hoc comparisons served as supporting evidence for H2 if the mean of question 2 was higher than that of question 1. H3 was supported if the means of question 3 and 4 were higher than those of question 2 and 3. 

Single sample *t*-tests were conducted on two groups that answered two questions representing intrapersonal and interpersonal loss in study 2 to assure the extent of asymmetry of psychological reactions to gain and loss. Specifically, students were not loss-averse if the loss aversion ratio was not significantly different from 1 or significantly below 1. In contrast, they were slightly averse to loss if the loss aversion ratio was significantly higher than 1 but significantly lower than 2. This served as supporting evidence for H4. Similarly, typical loss aversion occurred if the loss aversion ratio was significantly higher than 1 and was not significantly different from 2. This served as supporting evidence for H5. Finally, independent sample ANOVA was used to test whether students were more averse to interpersonal loss than intrapersonal loss. H6 was supported if the mean of the interpersonal condition was significantly higher than that of the intrapersonal condition.

## 3. Results

### 3.1. Sensitivity to Changes in Points

Table 1 shows descriptive statistics and intercorrelations from study 1. In question 1, the students’ average rating was 3.39, which indicated that they felt slightly satisfied with the intrapersonal 5-point gain in subject A. However, they became slightly dissatisfied with their performance (the average rating declined to 2.61) when the identical intrapersonal 5-point gain was accompanied by an intrapersonal 5-point loss (from 80 to 75 points in subject B). Theoretically, the amount of intrapersonal loss in question 3 was identical to that of question 2, as the average points of both questions were the same (82.5). Consequently, no difference should have emerged in satisfaction levels between the two questions. However, the average rating for satisfaction level rose to 3.10 when an identical intrapersonal 5-point loss (from 80 to 75 points) was accompanied by an interpersonal 5-point gain (individual 75 points was 5 points higher than the class average). The rating rose even higher, to 3.93 when the same intrapersonal loss (from 80 to 75 points) was accompanied by a relative interpersonal 10-point gain (the individual 75 point score was 10 points higher than the class average). 

The test indicated that the main effect of question frames was significant (*F* = 33.83, *p* = 0.00, Cohen’s *f*^2^ = 0.30 indicated moderate effect size according to Cohen (1988)’s guidelines). As a result, H1 was confirmed, and students were shown to be more sensitive to changes than to their final grade. Post hoc comparisons indicated that mean differences among all the conditions were also significant (*p* value ranged from 0.00 to 0.01). Specifically, the mean difference of −0.78 was calculated by subtracting the mean of question 1 (3.39) from that of question 2 (2.61). This served as supporting evidence for H2; it was found that students felt a bit dissatisfied when their current performance was inferior to their normal level. The mean differences were calculated by subtracting the mean of question 2 (2.61) from that of question 3 (3.10) and by subtracting the mean of question 3 (3.10) from that of question 4 (3.93) and were 0.49 and 1.32, respectively. This served as supporting evidence for H3 and showed that the feelings of dissatisfaction that students felt as a result of their intrapersonal loss could be compensated and even overturned by an increasing amount of interpersonal gain.

### 3.2. Aversion to Intrapersonal and Interpersonal Losses 

Table 2 shows the results of study 2. The average intrapersonal loss aversion ratio (*M* = 0.31, *SD* = 0.84) was significantly lower than 1 (*t* = −4.95.36, *M*_D_ is −0.69, 95% CI = [−0.97, −0.41], *p* = 0.00) and 2 (*t* = −12.32, *M*_D_ is −1.89, 95% CI = [−2.20, −1.58], *p* = 0.00). H4 was not supported by these results, which indicated that students were not averse to intrapersonal loss. In addition, the average interpersonal loss aversion ratio (*M* = 1.76, *SD* = 0.99) was significantly higher than 1 (*t* = 4.69, *M*_D_ is 0.76, 95% CI = [−0.43, 1.10], *p* = 0.00), and it was not significantly different from 2 (*t* = −1.45, *M*_D_ is −0.24, 95% CI = [−0.57, 0.10], *p* = 0.16). This indicates that students were averse to interpersonal loss and that the extent of loss aversion ratio was identical to typical findings in the Economics field. Consequently, H5 was also supported.

Finally, the main effect in the independent sample ANOVA showed that, compared to the intrapersonal loss condition, the average loss aversion ratio was significantly higher in the interpersonal loss condition (*F* = 45.16, *p* = 0.00, Cohen’s *f*^2^ = 0.64, which indicated a large effect size according to Cohen’s guidelines (1988)). As a result, H6 was confirmed, and it was found that students were so afraid of being judged as incompetent that they were more averse to interpersonal loss.

## 4. Discussion and Implications

The present study proposed an alternative measure that differed from those typically used in educational research and sought to frame the dynamic process of achievement motivation by focusing on loss aversion. It was found that college students were more sensitive to delineated changes in performance than to their final performance. Specifically, the level of satisfaction that students felt with their final grade (set to 82.5 points) varied across the four questions in study 1, even though the score was the same. Their satisfaction declined when their actual performance was 5 points lower than their normal level and then rose when the same intrapersonal loss was accompanied by an increasing amount of interpersonal gain. The results from study 1 thus implied the following: (1) measurements used in the achievement motivation (i.e., achievement goal) literature and delineated certain final states (i.e., to perform better on exams than I have done in the past) may be limited in their ability to accurately reflect students’ psychological reactions; (2) the discrepancy between the actual self and ought self (actual self was inferior to ought self) can cause dissatisfaction, which was consistent with the prediction of the self-discrepancy theory [10,15,16,17,18], but contradicted a recent related finding [11]; and (3) students felt proud and satisfied when their current performance was superior to others despite the fact that it fell below their normal level of performance, which supported the idea that the performance-approach goal proposed in the achievement goal theory may promote or be related to some positive affects [12,19,20,21,22].

Furthermore, it was found that students were not averse to intrapersonal loss but were significantly more averse to interpersonal loss. This indicates that if a student’s current performance was lower than expected, they did not experience loss aversion; the negative feelings linked to interpersonal loss could not be compensated by corresponding intrapersonal gain. Approximately twice the amount of intrapersonal gain is needed to compensate for interpersonal loss. The results of study 1 implied three findings. First, the discrepancy between actual self and ideal self did not cause negative effects, which contradicted to the prediction of the self-discrepancy theory and its empirical findings [10,11,16]. Second, the psychological reactions to gain or success and to loss or failure were asymmetrical, but this asymmetry was different when different sources of loss were involved (intrapersonal or interpersonal loss). Specifically, the psychological reaction to intrapersonal loss was less significant than it was to intrapersonal gain. In contrast, the psychological reaction to interpersonal loss was greater than it was to interpersonal gain. It may suggest that in the context of achievement goal, the approach-based goal (i.e., mastery-approach) and avoidance-based goal (i.e., mastery-avoidance) should be considered simultaneously in theory (i.e., multiple goal framework) [23] and in analysis (i.e., person-based analysis, such as latent profile analysis). Moreover, a mastery-approach goal (or self-approach goal) may be given less weight than a mastery-avoidance goal (or self-avoidance goal). On the other hand, a performance-approach goal may be given greater weight than a performance-avoidance goal. Third, students were only averse to interpersonal loss, which supported the finding that students feared being judged as incompetent [12,24,25]. However, loss aversion in the prospect theory was only partially supported because students were only averse to interpersonal loss. 

In practice, the above results suggest that there may be no significant effect when teachers explicitly encourage intrapersonal comparisons in students (i.e., set what they have done in the past as a standard for comparison to their current or future performance). In contrast, teachers can moderately integrate implicit competition during teaching and learning activities (e.g., privately provide individuals with information regarding the normal level of mastering a certain concept, or normative performances on certain subject learning or related exam), rather than actively or overtly initiate success and failure resulting from interpersonal comparisons (i.e., explicit competition). 

## 5. Conclusions

This study demonstrated that college students were more sensitive to performance changes than to their current or final performance and that they were averse to interpersonal loss but not intrapersonal loss. This may imply that avoidance motivation was stronger than approach motivation. In addition, a few findings were inconsistent with a theory that was tested using similar and different instruments. The inconsistency between the findings of the present study and the prospect theory can help explain the boundary of the latter in the achievement context. The inconsistency between the findings of this study and the self-discrepancy theory may imply that the present measurement served as an effective alternative to traditional measurement (i.e., questionnaires) for eliciting students’ psychological reactions to gain and loss in the achievement context. However, more research, using the same or similar measurements and a larger sample size, is needed to further examine the findings of this study. It also important that researchers investigate the effects of applying the present findings to real-life instances of learning performance assessment. Future studies are encouraged to use individuals’ actual performance as reference points to re-examine present findings, and it will be helpful to clarify if individual motivational (e.g., self-efficacy), demographic (e.g., gender, socioeconomic status), environmental (e.g., parenting style), and instructional factors (e.g., lassroom climate, teaching style, and test frequency) influence an individual’s loss aversion, which could be confounding study results.

## Figures and Tables

**Table 1:** Study 1: Descriptive statistics and intercorrelations.

Conditions and Goals	*M*(*SD*)	1	2	3	4
1 intrapersonal 5 points gain	3.39(1.02)	—			
2 intrapersonal 5 points gain, 5 points loss	2.61(0.77)	0.61 **	—		
3 intrapersonal 5 points loss, interpersonal 5 points gain	3.10(0.86)	0.64 **	0.47 **	-	
4 intrapersonal 5 points loss, interpersonal 10 points gain	3.93(0.88)	0.48 **	0.25	0.74 **	—

** *p* < 0.01.

**Table 2:** Differences in loss aversion ratio for intrapersonal and interpersonal loss condition.

Measure	Loss Aversion Ratio		*F*(1, 70)	*f* ^2^
	*M*	*SD*		
1 intrapersonal 5 points gain and 5 points loss	0.31	0.84	45.16 ***	0.64
2 intrapersonal 5 points gain, interpersonal 5 points loss	1.76	0.99		

*** *p* < 0.001.

## Data Availability

The data presented in this study are available on request from the corresponding author. The data are not publicly available due to privacy and ethic concerns.
